# REM Sleep Loss-Induced Elevated Noradrenaline Plays a Significant Role in Neurodegeneration: Synthesis of Findings to Propose a Possible Mechanism of Action from Molecule to Patho-Physiological Changes

**DOI:** 10.3390/brainsci14010008

**Published:** 2023-12-21

**Authors:** Shatrunjai Giri, Rachna Mehta, Birendra Nath Mallick

**Affiliations:** 1 Department of Biosciences, Manipal University Jaipur, Jaipur 303007, India; shatrunjai.giri@jaipur.manipal.edu; 2 Amity Institute of Neuropsychology & Neurosciences, Amity University, Noida 201301, India; rmehta2@amity.edu

**Keywords:** memory consolidation, neurodegeneration, noradrenaline, REMS loss, synaptogenesis

## Abstract

Wear and tear are natural processes for all living and non-living bodies. All living cells and organisms are metabolically active to generate energy for their routine needs, including for survival. In the process, the cells are exposed to oxidative load, metabolic waste, and bye-products. In an organ, the living non-neuronal cells divide and replenish the lost or damaged cells; however, as neuronal cells normally do not divide, they need special feature(s) for their protection, survival, and sustenance for normal functioning of the brain. The neurons grow and branch as axons and dendrites, which contribute to the formation of synapses with near and far neurons, the basic scaffold for complex brain functions. It is necessary that one or more basic and instinct physiological process(es) (functions) is likely to contribute to the protection of the neurons and maintenance of the synapses. It is known that rapid eye movement sleep (REMS), an autonomic instinct behavior, maintains brain functioning including learning and memory and its loss causes dysfunctions. In this review we correlate the role of REMS and its loss in synaptogenesis, memory consolidation, and neuronal degeneration. Further, as a mechanism of action, we will show that REMS maintains noradrenaline (NA) at a low level, which protects neurons from oxidative damage and maintains neuronal growth and synaptogenesis. However, upon REMS loss, the level of NA increases, which withdraws protection and causes apoptosis and loss of synapses and neurons. We propose that the latter possibly causes REMS loss associated neurodegenerative diseases and associated symptoms.

## 1. Introduction

Nerve cells possess the extraordinary capability of integration of signals as well as long-distance, direct, and faster intercellular communication. These are essential for brain functioning and maintenance of faster and precise homeostasis of the whole organism as a system. Since matured neurons normally do not possess the capacity for cell division and regeneration, any type of external or internal insult may cause loss of neurons and synapses (neurodegeneration), leading to a breakdown in signal processing and communication. Neurodegeneration precedes neurological disorders, the probability of which exponentially increases with predisposing factors which include ageing, injury, psycho-somatic diseases, unhealthy lifestyle, etc. As the age progresses, the homeostatic stress-tolerance mechanism tends to become disbalanced and fails to bear the load of disease-related stress molecules (if any), which predisposes progressive neurodegeneration [1]. Pathological protein aggregation, synaptic and neuronal network dysfunction, oxidative stress, cytoskeletal abnormalities, altered energy metabolism, and apoptosis are the various hallmarks of neurodegeneration and associated dysfunctions [2,3]. These may cause memory impairment, cognitive disabilities, behavioral abnormalities, and sensory and/or motor function loss. The structural integrity of neurons, particularly in relation to their dendritic arborizations, as well as synaptic contacts with the target neurons play a crucial role in appropriate functioning of the brain and the nervous system. Such integrity is maintained by metabolic processes, while factors including hormones, neurotransmitters, and behavior affect them directly and/or indirectly. Targeting the desired neurons is the fundamental property for optimum functioning of the brain including learning, memory consolidation, and its retrieval. The fundamental processes including learning, memory consolidation, and its retrieval are essential for optimum functioning of the brain that leads to execution of basic functions for survival of the body as a system. It is, therefore, proposed that it is quite likely that at least an inherent and instinctive physiological process primarily regulated by the brain, of the brain, and for the brain must modulate neuronal survival and synaptic integrity. One such instinctive physiological process expressed by all living beings (at least higher in evolution) is sleep-wakefulness, whose role in neuronal survivability and integrity have been addressed below.

### 1.1. Sleep and Wakefulness

Basic rest and activity are alternatively and non-rhythmically expressed by all living organisms. As they are expressed non-rhythmically, it suggests that they are likely to be modulated by local, intrinsic, and extrinsic factors. Apparently, these states have often been defined and identified by the presence or absence of expression of active physical movement of the organism(s). However, with the evolution of species, as the brain evolved, it was a challenge to differentiate the rest taken by the physical body from that of the rest taken by the brain. Although the rest taken by the body could be identified by the apparent loss of physical movement, for the brain, the primary limitation was to objectively define its active and inactive (non-active) states and achieving this non-invasively. Subsequently, in the absence of any other parameter, one turned to the conscious states. As wakefulness was often associated with physical movement, while sleep with physical rest, the latter was considered as rest for the brain. However, in the absence of any objectively defining characteristic, it was not possible to differentiate physical rest from that of sleep, which is more than just physical rest. Added to that, one had the experience of intermittently dreaming during sleep. The breakthrough was possible after the discovery of electroencephalogram (EEG) and its correlation with the states of consciousness, sleep, and wakefulness. After such discovery of EEG, it was identified that sleep is not a homogeneous state. It was identified that, within sleep, the brain waves intermittently appear to be apparently comparable to that of wakefulness, and that often dreams appear during such an active stage. This led to classifying the sleep stage into rapid eye movement sleep (REMS) and non-REMS.

### 1.2. Rapid Eye Movement Sleep (REMS)

REMS was discovered in 1953 [4] and, like many other discoveries, this was also a serendipitous identification in humans. Although it was identified in animals later [5], some earlier studies had mentioned some of the associated symptoms, which retrospectively help understand brain regulation of REMS [6,7]. Based on electrophysiological signals from the brain, eye movements, and muscle activity, the modern-day science has classified consciousness in the animals higher in evolution including humans broadly into three contiguous states viz. wakefulness, sleep (non-REMS), and REMS. In fact, identification of REMS had put an end to the argument that sleep is a homogenous and passive phenomenon. We must confess that in the absence of an organized and evolved brain, we cannot comment on the existence of REMS state and consciousness in the lower species. Another interesting attribute of REMS is that often (though not exclusively) dreams are associated with this stage of sleep [8].

### 1.3. REMS in Different Animal Species

In many species including humans, among the three major accepted conscious states (wakefulness, sleep, and REMS), although one spends the least duration in REMS, it appears to have been conserved through evolution [9,10,11]. Qualitatively, REMS is expressed throughout an individual’s life. Although its quantity reduces from birth, to neonatal, to adulthood, and to old age, it is never absent [12,13]. Many authors have attempted to find a correlation between the amount of REMS with various variable parameters e.g., body size or weight [14], brain size [11], body or brain temperature [15,16], natural habitat [17,18], etc. Although one may find some loose correlation among a few of those variables (factors), an unambiguous and strong correlation was missing. Notwithstanding, the importance or functions or contribution of REMS in maintaining normal physiological processes have been proposed based on REMS deprivation (REMSD) studies.

### 1.4. Neural Regulation of REMS by the Brain

REMS is autonomically regulated. Like the regulation of many other instinct functions and related behaviors regulated by the brain, the basic neuronal scaffold for REMS regulation is also located in the brainstem, which, however, may be modulated by the host of areas in the brain, hormonal systems, peripheral inputs, etc. [19,20,21,22,23,24]. REMS is modulated in almost all acute as well as chronic disorders [25,26,27], and, depending on chronicity of its disturbance, almost all physiological processes are negatively affected. Its disturbance during sleep is often termed as REMS behavioral disorder, while subject to confirmation, its disorder during wakefulness has often been compared with hallucination, psychological derangements, day dreaming, etc. [28]. Under normal conditions, REMS usually does not appear during wakefulness, while some amount of non-REMS is a pre-requisite for the appearance of REMS [29,30]. Therefore, in principle, although REMS loss may be possible without significantly affecting other states, loss of non-REMS does not allow for the appearance of REMS. Thus, the loss of non-REMS essentially and for all practical purposes leads to loss of both REMS and non-REMS, i.e., loss of total sleep. Indeed, most experimental studies have been conducted by restricting partial or complete loss of REMS (when, depending on conditions, there may be some loss of non-REMS) and a few upon total sleep loss (i.e., upon loss of both, non-REMS as well as REMS).

### 1.5. Functions of REMS

REMS is necessary for leading a normal life. Its importance may be underscored by the fact that loss of REMS is compensated by a rebound increase during recovery from REMS loss and its sustained loss becomes fatal [31]. It is affected to various degrees in almost all acute and chronic diseases. In other words, there is hardly any disorder where REMS is immune from being affected [32,33]. On the other hand, REMSD affects most physiological and psycho-somatic processes including for example, cardiovascular and respiratory systems [34,35,36,37], metabolic processes [38], endocrine system [39,40], psycho-somatic–behavioral processes [33,41,42], and so on. REMS plays an important role in supporting healthy living by regulating the cellular, molecular, and metabolic processes [38] including clearing out toxins and waste products [43,44]. It may be pertinent to mention here that, for convenience of explaining the latter, we have proposed a term ***hypnoclean*** [45]. It has been proposed that REMS has evolved to maintain the optimum level of noradrenaline (NA) in the brain [46], which in turn maintains neuronal integrity, excitability, and house-keeping function of the brain [47]. As neurons are terminally differentiated cells, their loss including degeneration is usually irreversible. However, before the degeneration sets in, the neurons (any cell for that matter) are likely to express some early signs, which may serve as indicator to initiate preventive action(s).

In this write-up, we will first correlate neurodegenerative changes with REMS disorders and then identify the REMS-loss associated factor(s) responsible for such degenerative changes. It is a fact that REMS or its loss is a complex behavioral expression, which is associated with an equally complex underlying changes in levels of biomolecules regulating its expression resulting in homeostatic disbalance. We hypothesized that fundamentally one or more factor(s) responsible for the regulation of REMS regulation must become disbalanced to cause REMS dysfunction, which then plays a major role in causing the REMS-loss associated changes. Therefore, we will briefly discuss the neuronal and neurochemical regulation of REMS and identify possible crucial biomolecule(s) affected during REMS dysfunction. Thereafter, we will correlate if and how such biomolecules play any significant role in maintenance of neuronal integrity, degeneration, synaptogenesis or synaptic loss, and apoptosis, which ultimately affect neuronal and brain functions.

### 1.6. Noradrenaline Level in Relation to REMS and Its Loss

It is well known that functional reciprocal interaction between the NA-ergic REM-OFF neurons in locus coeruleus (LC) and the cholinergic REM-ON neurons in the pedunculo-pontine tegmentum (PPT) essentially form the basic neuronal scaffold for the regulation of REMS [24,48]. Further, the GABA-ergic interneurons and presynaptic inputs in the LC are necessary for inhibition and disinhibition of the neurons and regulate REMS [49,50]. Most of the NA-ergic neurons in the LC are REM-OFF type. Normally, these neurons are rhythmically active and cease activity during REMS [51], while they continue activity during REMSD [52]. Based on such information it was proposed and subsequently confirmed that the level of NA in the brain would be minimum during REMS, while it would be highest upon REMSD [53]. As a corollary, it was then proposed that REMSD-associated symptoms could be mediated by the elevated level of the NA in the brain. However, the challenge was to understand how the elevated NA could be responsible for both the acute as well as the chronic symptoms and disorders. By carefully reviewing and analyzing the available literature from various related fields including those contributed by us, it was proposed that, for acute symptoms, NA could affect neuronal excitability, while in addition for chronic effects, it could affect either the gene/transcription level or neuronal survival. Findings from isolated studies indeed supported the said contentions, e.g., REMSD-associated elevated NA increased the Na-K ATPase activity, one of the major underlying causes for maintenance of brain excitability [47]. Subsequently, it has been shown that the NA may affect Na-K ATPase gene regulation supporting the possibility of a sustained effect on brain excitability [54]. These findings, although supported mostly the acute symptoms and some sustained behavioral symptoms, require evidence to support the REMSD-associated neurodegenerative changes associated with chronic disorders. Before we proceed further, we will first review the knowledge available to show that REMS loss has been correlated with memory loss and neurodegenerative diseases.

## 2. REMS Loss and Neurodegenerative Diseases: Role of Noradrenaline

Neurodegenerative diseases, such as Alzheimer’s, Parkinson’s, and Huntington’s diseases, are usually accompanied by disturbances in sleep–wake cycles and circadian rhythm. However, sleep disruptions in these conditions are frequently underestimated by patients and caregivers, and healthcare professionals may overlook their significance. The impact of disrupted sleep on patients’ quality of life is significant and can also pose safety risks. Interestingly, it is believed, retrospectively, that sleep dysfunctions may appear much before the onset of these disorders, sometimes years or decades in advance. For instance, REMS Behavior Disorder (RBD) could be a notable early sign of synuclein-specific neurodegenerative disorders, while excessive daytime sleepiness has been proposed to be associated with the development of Parkinson’s disease [55,56].

### 2.1. Alzheimer’s Disease (AD)

Sleep and circadian-rhythm disruptions have long been associated with Alzheimer’s disease (AD), the most prevalent neurodegenerative disorder. Recent evidence suggests a two-way relationship between AD and sleep/circadian-rhythm disturbances [57]. Aging-related sleep changes are more pronounced in individuals with AD, leading to fragmented sleep, reduced total sleep time, increased sleepiness, frequent napping, and behavioral changes known as sundown syndrome. These alterations not only impact patients but also place significant strain on their family members and caregivers [58]. In AD, there are specific changes in sleep patterns, including fewer and poorly formed spindles and K complexes, decreased slow-wave sleep, and alterations in REMS characteristics [59,60,61]. Recently, the role of obstructive sleep apnea (OSA), which has been associated with REMS loss and disturbance, has been recognized as a causative or predisposing factor for cognitive impairments among AD patients [62,63]. Disturbed sleep patterns have also been linked with increased risk of developing AD, cognitive impairment, and preclinical AD [64,65]. Advanced biomarkers have enabled a more detailed analysis of the relationship between sleep and AD, revealing that disruption of REMS is associated with higher levels of beta-amyloid, a protein implicated in AD [66,67]. Tau aggregation, another hallmark of AD, is also modulated by the changes in the sleep/wake cycle [68,69]. Longitudinal studies have identified changes in specific EEG markers as predictors of the rate of beta-amyloid accumulation over several years [70,71].

Furthermore, alterations in REMS, such as reduced REMS duration and longer REMS latency, have been associated with dementia development [65,72,73]. The glymphatic system, responsible for clearing chemical waste from the brain, operates primarily during sleep and is suppressed during wakefulness [74]. Disrupted sleep or REMS may impede the clearance of amyloid beta and tau proteins by the glymphatic system [75,76]. Further, NA, which affects REMS, is also an important factor that affects glymphatic system. There are two aspects to consider regarding the role of NA in relation to the glymphatic system and the extracellular perivascular space. First, during sleep, the declined level of NA causes expansion in the perivascular space surrounding blood vessels, which causes decreased resistance to fluid transport and an increased rate of glymphatic clearance [77]. This is reflected by improved cerebrospinal fluid (CSF) infiltration and increased interstitial solute clearance [78]. Second, during arousal, NA is released in bursts leading to inhibition of the activity of the glymphatic system and increased resistance to fluid transport [79]. Also, it has been suggested that hyperphosphorylated neurofibrillary tangles in LC may be responsible for NA-ergic axonal degeneration and cell death in AD patients [80,81] and in animal model [82]. These might contribute to impaired NA neurotransmission to the hippocampus causing impaired cognition and dementia in this disease [83]. Proper assessment of sleep disturbances in AD often requires interviewing caregivers, as patients may not be aware of these issues or they may be unable to explain properly. Evidence suggests that good sleep hygiene practices can improve sleep quality in AD patients, with potential cognitive benefits [84].

### 2.2. Parkinson’s Disease (PD)

In 1817, James Parkinson first described disrupted sleep in his initial clinical depiction of Parkinson’s disease (PD). However, a systematic examination of the impact of sleep disturbances and alertness in PD has only been conducted in the past few decades. Most PD patients experience excessive daytime sleepiness and/or disrupted nighttime sleep [85]. Polysomnographic studies have revealed that up to 80% of PD patients report poor sleep quality, characterized by prolonged sleep latency, fragmented sleep, and reduced REMS [85,86,87].

RBD is characterized by REMS associated dream enactment behaviors. It is associated with up to 60% of PD patients and serves as a significant prior manifestation of PD and other synucleinopathies, e.g., dementia with Lewy bodies and multiple system atrophy [88]. Excessive daytime sleepiness gained attention in the early 1990s. It is more prevalent in PD compared to other chronic diseases and pose the highest risk for its development [89,90]. Unique mechanisms underlie disturbed alertness in PD, including loss of hypocretin neurons, degeneration of neurons in the wake-promoting centers and their projections to the brainstem, and circadian disruption [91,92]. Furthermore, there is some evidence of NA-ergic system impairments involved in the cognitive and emotional deficits in PD. Loss of NA-ergic neurons has been reported to be due to accumulation of α-synuclein in the LC leading to a decreased NA level and degeneration of the nigrostriatal pathway [93]. As a result, it has been suggested that, possibly due to the lack of NA, there is withdrawal of neuroprotective effects leading to neurodegeneration [94].

In conclusion, disturbances in sleep and alertness in PD present a complex landscape. Although several diagnostic and conditional or symptomatic treatment approaches have been discussed in many reviews [95,96], there is a need and scope for further research to develop and optimize PD-specific diagnostic tools particularly in relation to sleep disorders. Effective evidence-based treatments for sleep dysfunction in PD are currently lacking, emphasizing the importance of clinical investigations into sleep therapeutics for PD [97]. Targeting sleep may have beneficial effects on both motor and non-motor symptoms of PD and potentially impact disease progression. Therefore, we propose that estimation of sleep loss may be considered to serve as a prognostic or predictive value in PD.

### 2.3. Huntington’s Disease

Huntington’s disease (HD) is a progressive neurodegenerative disorder characterized by movement difficulties, cognitive decline, and psychiatric symptoms. Sleep disturbances are commonly experienced by individuals with HD, affecting up to 90% of patients. However, compared to other neurodegenerative diseases, sleep disturbance in HD have not received as much comprehensive attention and investigation [98]. Despite being associated with the severity and duration of the disease, disturbed sleep has been reported even in the early stages of HD, including among asymptomatic carriers of the HD mutation [99,100]. Polysomnographic studies have shown that sleep in HD is often fragmented, with a reduced NREM-3 stage and increased sleep spindle density [101]. While some studies indicate a reduction in REMS in HD, this finding has not been consistently replicated [101,102]. RBD has also been reported in HD and associated depression and neuropsychiatric manifestations [103]. This highlights the importance of timely diagnosis and effective treatment of sleep disturbances in HD patients.

The diagnosis of sleep and wake disorders in the HD population relies on standard methods and tests used in general sleep medicine. However, the complexities of HD that influence sleep patterns necessitate the development of new assessment methods for sleep and alertness specifically tailored for HD. There is a lack of therapeutic studies specifically focusing on sleep dysfunction in HD. Additionally, managing sleep problems in HD is further complicated by complex medication regimens, some of which may have negative effects on sleep and daytime alertness. Non-pharmacological approaches, such as physical exercise, hold promise as a potential strategy for managing sleep dysfunction in HD [104]. Therefore, it can be concluded that neurodegenerative changes are strongly correlated with REMS loss and its associated disorders.

## 3. Risk Factors of Neurodegeneration and REMS Loss

Some common normal or lifestyle has been proposed to act as risk or predisposing factors associated with neurodegenerative disorders; however, their cause-and-effect relationship and mechanism of action are yet unknown. As most lifestyle and related risk factors affect a common fundamental physiological process, sleep, we argue that it is possible that such sleep changes might affect levels of NA in the brain and, in turn, cause neurodegeneration and associated diseases.

### 3.1. Age

Advancing age is among the primary risk factors associated with neurodegenerative diseases [105]; however, all aged persons do not get affected identically [106]. One common condition often observed among the aged persons is a sustained decline in sleep quality [107,108,109]. A correlation among aging, a reduction in REMS [109], and an increased incidence of neurodegenerative diseases like AD [110,111], PD [110,112], and Huntington’s [113,114] diseases have been reported. Additionally, a study conducted on rodents [115] discovered that sleep deprivation in early life poses a significant threat to neural health in later stages, underscoring the necessity of comprehending the long-term consequences of chronic sleep restriction in humans. Furthermore, it has been observed that NA levels are elevated in older adults compared to younger individuals, not only in the brain but also in other body fluids such as plasma [116,117,118]. Other studies have shown that subcortical NA levels are approximately twice as high in healthy older adults compared to younger individuals. It has been observed that the age-related increase in NA levels in the heart and plasma tends to rise by approximately 10–15% per decade of aging [119,120,121,122]. Elevated NA levels have also been associated with hyperphosphorylation of tau protein, resulting in an increased formation of tau tangles and amyloid plaques, a characteristic feature of AD [123,124]. Drugs that lower NA levels have been reported to reduce the risk of various diseases, including AD [125]. As mentioned above, we have shown that loss of REMS increases NA levels in the brain and causes neurodegeneration. Therefore, it is plausible that REMS-loss-associated elevated NA could contribute to the health disorders mentioned above.

### 3.2. Genetic and Epigenetic Changes

Some neurodegenerative diseases have a genetic component, wherein specific genetic mutations or variations contribute to an increased susceptibility to these disorders. For instance, familial forms of AD have been linked to mutations in genes such as amyloid protein precursor, presenilin 1, and presenilin 2, while HD is caused by mutations in the huntingtin gene. Interestingly, sleep disturbances have been implicated in exacerbating the effects of these genetic mutations and promoting neurodegeneration [126]. In AD, mutation in the amyloid protein precursor alters the levels of amyloid beta-42 (Aβ42) protein and renders it susceptible for aggregation, thereby causing AD [127]. The aggregation of Aβ42 has been found to be increased upon sleep including REMS loss, which suggests potential correlation between chronic sleep loss and pathogenesis of AD [128,129]. Similarly, progression of PD is also susceptible to sleep-loss disorders, and vice versa [130]. A study has shown that RBD could accelerate the neurodegeneration and compromise mental functioning in PD patients [131]. RBD has been reported in familial neurodegenerative conditions such as parkinsonism related to parkin and DJ-1 mutations, spinocerebellar ataxia type III, and amyotrophic lateral sclerosis (ALS) due to SOD1 mutations [132].

Recent evidence has shown that epigenetic mechanisms, particularly chromatin acetylation, are involved in the regulation of cognitive functions [133,134]. It has been observed that the expressions of histone acetyl transferases (HATs) and their activities are reduced upon REMSD. Reduced expression and function of HATs may lead to neuronal dysfunction associated with decreased histone acetylation as is observed in several mouse models of neurodegenerative diseases and disrupted sleep–wake cycles in flies [135,136]. Another study found increased HDAC protein levels in REMS-deprived rat brain, which further deacetylated histones leading to impaired learning and memory [137]. In our study we have reported changes in gene expression upon REMSD [138], which, in turn, might affect the neurodegenerative diseases.

### 3.3. Family History

Having a family history of neurodegenerative diseases increases the risk of developing the same or a related disorder [132,139]. For example, in the case of ALS, a complex multisystem disorder including cognitive deficit and extrapyramidal dysfunction has been reported to have a positive family history in up to 10% of patients with association with causative gene mutations [139]. Clinical studies have demonstrated that a positive family history of ALS is a risk factor for the development of ALS [140,141]. On the other hand, as a critical argument, although RBD has been reported in familial neurodegenerative conditions such as PD, critical studies are needed to systematically assess the possible heritability and relationship with sleep or REMS loss, if at all [132].

### 3.4. Environmental Factors

Exposure to certain environmental factors has been implicated with prevalence of neurodegenerative diseases [142]. Air pollution has been linked to neurodegenerative disorders such as AD [143] and PD [144,145]. Air contaminants are the primary cause of lung and brain inflammation, which disrupt the proper functioning of the central nervous system (CNS) [146]. Atmospheric pollutants, e.g., O_3_, SO_2_, NO_2_, CO, Pb, and, particulate matters (PM_2.5_, PM_10_), cause CNS pathology by causing oxidative stress, microglial cell activation, neuronal inflammation, and changes in blood–brain membrane permeability [147]. Metals like copper, iron, zinc, and aluminum potentially play a significant role as factors/cofactors in the etiology of various neurological disorders including AD for instance [143]. Heavy metals including lead, cadmium, arsenic, and manganese possibly lead to neurological-disease-like conditions resembling PD, AD by elevating neuronal oxidative stress, mitochondrial dysfunction, inflammatory processes, and apoptosis [148,149]. It may be reiterated, as mentioned above, that all the changes can be seen after REMSD. Further, although these heavy metals and environmental pollutants may affect sleep including REMS, whether they contribute to neurodegeneration directly or through changes in sleep needs detailed investigations.

### 3.5. Lifestyle Factors

Healthy, low-risk lifestyles (e.g., no smoking, daily exercise, moderate alcohol consumption, and having a moderate weight) have been correlated with significantly lower risk of neurodegenerative diseases than the high-risk lifestyle group [150]. On the other hand, most of the altered lifestyle factors, e.g., stress, lack of physical exercise, unhealthy nutrition, obesity, high serum triglycerides and higher serum cholesterol levels, smoking, alcoholism, and associated diseases have been correlated with sleep disturbance or sleep fragmentation [151,152,153,154,155,156,157]. Such poor sleep hygiene, as compared to individuals having quality sleep, may predispose oneself to higher rate of psychiatric disorders such as depression, which may cause neurodegenerative disorders such as AD and PD. Disturbed REMS has been often reported in cases associated with many of the high-risk lifestyle practices described above [158,159,160]. On the other hand, diet is one of the lifestyle factors that influence AD [161] and PD [162]; hyperlipidemic diet may promote β-amyloid deposition [163]. Interestingly, excess calorie intake or calorie deprivation has been shown to reduce the REMS episodes [151,152]. Calorie consumption, caffeine, alcohol, and metals absorbed through food and lipids, among other factors, have been linked to AD-related parameters such as epigenetic, β-amyloid, tau proteins, oxidative stress, and reactive oxygen species [164]. Furthermore, several research groups have highlighted the important role of NA in social behavior, intergroup relations, mood disorders, and moral decision-making [165]. Higher NA level has been found to negatively affect both motivation and social play [166] and is suggested to affect basic primary emotional arousal such as fear and aggression.

## 4. REMS, Its Loss, and Neuronal Degeneration

Neurodegeneration refers to progressive loss of structure or function of neurons in the central and/or peripheral nervous system. Depending on the neuron(s) affected in brain region, this will lead to the decline or impairment of cognitive and sensory–motor functions. It is a characteristic feature of various neurological disorders, such as AD, PD, HD, and ALS. Either total sleep loss or only REMS loss, even for 12 h, may provoke cognitive impairments [167]. A wide range of empirical evidence demonstrates impairments in memory, learning, attention, decision making, and emotional reactivity in healthy human subjects after sleep loss [168,169,170]. REMS loss impairs brain development, memory consolidation, and cognitive ability, while REMS improves most of those functions. Several studies around the globe including us (in animal models) have shown that REMSD leads to poor cognitive ability [171,172], impaired learning and memory [173,174] as well as neuroinflammation [45,175], neuronal apoptosis [176,177], damaged neuronal plasticity [178], and neuronal morphology [178,179,180,181]. REMSD affects CA1 cholinergic muscarinic receptors, which may be responsible for REMSD-associated amnesia [182]. Also, REMSD has been reported to increase the number of TUNEL-stained (a sign of DNA fragmentation and apoptosis) neurons in the hippocampus [174]. Furthermore, REMSD has been shown to increase accumulation of toxic byproducts, e.g., amyloid β42, phosphorylated tau protein [74,183] in the brain; it also caused mitochondrial dysmorphism, activated caspase, and increased cytochrome release [176,184]. Accumulation of those factor(s) due to REMS loss could predispose and/or exacerbate neurodegeneration causing physiological deficits including impaired memory, and neurodegenerative disorders.

### Elevated Level of NA and Neurodegeneration

Previous studies have shown that REMSD-associated elevated level of NA leads to acute as well as chronic effects including increased excitability and irritability [47], anxiety [185,186], memory deficits [187,188], neuronal cytomorphological changes [180,181], apoptosis, and neuronal loss [177,180]. Subsequently, it has been shown that the REMSD associated elevated NA activated the mitochondria mediated intrinsic pathway to cause apoptosis and neuronal degeneration [176]. Our recent studies have shown that REMSD-associated altered level of NA impairs plasticity of the hippocampal neurons, which includes loss of neuronal arborization and dendritic spines, reduced synaptic proteins and ultrastructural parameters [178]. Upon careful analysis, it was observed that, upon REMSD, NA levels increased in all brain areas except the hippocampus [53]. Further, we have also reported that lower doses of NA promote neuronal branching, while its higher doses cause neuronal damage and disintegration [178]. Additionally, we have recently reported that lower doses of NA protect neurons from oxidative damage, whereas higher doses not only failed to provide protection, but also increased oxidative damage and neuronal apoptosis [46]. Collating all these findings from related, complementary, but isolated and independent studies, we propose that the hippocampus normally receives a lower (optimal) amount of NA, which protects neurons, facilitates branching, spine formation, and synaptogenesis, thus favoring learning and memory consolidation. However, upon experimental REMSD (i.e., reduced REMS), the level of NA decreases significantly (almost negligible) in the hippocampus. As metabolic processes leading to generation of oxidative load is a continuous process, the withdrawal of NA is likely to withdraw the protection from the neurons. This will compromise the processes of branching and synaptogenesis, which are essential for memory formation. On the other hand, in other areas of the brain where there will be an elevated level of NA, it will induce additional damage to the neurons as shown earlier that higher level of NA causes increased damage to the neurons [46,178].

Thus, upon REMS loss, be it either withdrawal of NA in the hippocampus or elevated level of NA in other regions of the brain, both cause neuronal degeneration, but apparently via different molecular mechanisms. The above explanations support the role of reduced level of NA in association with normal REMS in memory consolidation, and increased level of NA during REMS loss with memory impairment and neurodegenerative disorders, e.g., AD [189], PD [190], and depression [191], etc. However, detailed molecular mechanism of action of low and high doses of NA in neuronal protection and degeneration, respectively, needs further detailed study.

## 5. Conclusions

**Figure 1:**
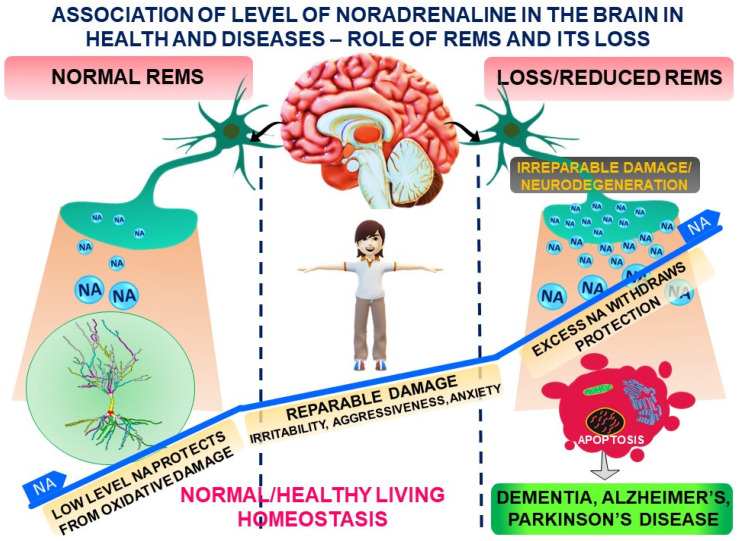
A schematic representation of altered levels of noradrenaline in brain as a possible factor for REMS-loss-associated brain dysfunctions and neurodegenerative disorder.

In conclusion, quality and healthy sleep practices appear to be associated with neuroprotective effect. Such practices could potentially slow down neurodegenerative disease progression and, thereby, reduce an individual’s risk of dementia and neuromotor disorders in later life. Neurodegeneration is a chronic effect, and most of the factors affecting lifestyle compromise sleep. We propose that lifestyle changes might facilitate neurodegeneration by inducing sleep disturbances and the effects are mediated by elevated level of NA in the brain (Figure 1). Interestingly, within limits the sleep loss associated effects may be reversed or recovered. Therefore, we propose that recording of history of sleep profile of patients must be considered as standard practice, which may help prevent irreversible damage at a later age and may have a significant prognostic value. Furthermore, the importance and role of sleep, its routine, and hygiene must be taught compulsorily from childhood. The beneficial effect of sleep and deleterious effect of sleep loss must be advertised aggressively as has been followed for role of exercise in maintenance of healthy heart and negative health effect of smoking, or drug abuse, or environmental pollution, respectively.

## Data Availability

Not applicable.

## Abbreviations

AD	Alzheimer’s disease
PD	Parkinson’s disease
GABA	Gamma-amino butyric acid
LC	Locus coeruleus
NA	Noradrenaline
NREMS	Non-REMS
RBD	REM sleep behavior disorder
REMS	Rapid eye movement sleep
REMSD	REMS deprivation
